# Chromosome-Scale Genome Assembly for Clubrush (*Bolboschoenus planiculmis*) Indicates a Karyotype with High Chromosome Number and Heterogeneous Centromere Distribution

**DOI:** 10.1093/gbe/evae039

**Published:** 2024-03-06

**Authors:** Yu Ning, Yang Li, Hai Yan Lin, En Ze Kang, Yu Xin Zhao, Shu Bin Dong, Yong Li, Xiao Fei Xia, Yi Fei Wang, Chun Yi Li

**Affiliations:** Wetland Research Center, Institute of Ecological Conservation and Restoration, Chinese Academy of Forestry, Beijing, China; Sichuan Zoige Wetland Ecosystem Research Station, Tibetan Autonomous Prefecture of Aba, China; Huzhou University, Huzhou, China; Institute of Information Technology, Chongqing Academy of Forestry Sciences, Chongqing, China; Wetland Research Center, Institute of Ecological Conservation and Restoration, Chinese Academy of Forestry, Beijing, China; Sichuan Zoige Wetland Ecosystem Research Station, Tibetan Autonomous Prefecture of Aba, China; College of Biological Sciences and Biotechnology, Beijing Forestry University, Beijing, China; College of Biological Sciences and Biotechnology, Beijing Forestry University, Beijing, China; Wetland Research Center, Institute of Ecological Conservation and Restoration, Chinese Academy of Forestry, Beijing, China; Sichuan Zoige Wetland Ecosystem Research Station, Tibetan Autonomous Prefecture of Aba, China; National Natural History Museum of China, Beijing, China; Wetland Research Center, Institute of Ecological Conservation and Restoration, Chinese Academy of Forestry, Beijing, China; Sichuan Zoige Wetland Ecosystem Research Station, Tibetan Autonomous Prefecture of Aba, China; Wetland Research Center, Institute of Ecological Conservation and Restoration, Chinese Academy of Forestry, Beijing, China

**Keywords:** *Bolboschoenus*, *Scirpus*, genome assembly, karyotype, holocentric chromosome

## Abstract

*Bolboschoenus planiculmis* (F.Schmidt) T.V.Egorova is a typical wetland plant in the species-rich Cyperaceae family. This species contributes prominently to carbon dynamics and trophic integration in wetland ecosystems. Previous studies have reported that the chromosomes of *B. planiculmis* are holocentric; i.e. they have kinetic activity along their entire length and carry multiple centromeres. This feature was suggested to lead to a rapid genome evolution through chromosomal fissions and fusions and participate to the diversification and ecological success of the *Bolboschoenus* genus. However, the specific mechanism remains uncertain, partly due to the scarcity of genetic information on *Bolboschoenus*. We present here the first chromosome-level genome assembly for *B. planiculmis*. Through the integration of high-quality long-read and short-read data, together with chromatin conformation using Hi-C technology, the ultimate genome assembly was 238.01 Mb with a contig N50 value of 3.61 Mb. Repetitive elements constituted 37.04% of the genome, and 18,760 protein-coding genes were predicted. The low proportion of long terminal repeat retrotransposons (∼9.62%) was similar to that reported for other Cyperaceae species. The *K_s_* (synonymous substitutions per synonymous site) distribution suggested no recent large-scale genome duplication in this genome. The haploid assembly contained a large number of 54 pseudochromosomes with a small mean size of 4.10 Mb, covering most of the karyotype. The results of centromere detection support that not all the chromosomes in *B. planiculmis* have multiple centromeres, indicating more efforts are needed to fully reveal the specific style of holocentricity in cyperids and its evolutionary significance.

**Significance**

*Bolboschoenus planiculmis* (clubrush), a typical wetland plant, has potential for practical applications in phytoremediation and wetland conservation. The deficiency in available genetic information limits insights on its ecological adaptability and chromosomal evolution. We present the first chromosome-level genome assembly for the species, with high continuity, completeness, and detailed annotation of protein-coding genes and repeated sequences. The assembly suggests that the majority of the karyotype is constituted by similarly small chromosomes and reveals a heterogeneous distribution of centromeres along chromosomes and among them.

## Introduction

**Fig. 1.**
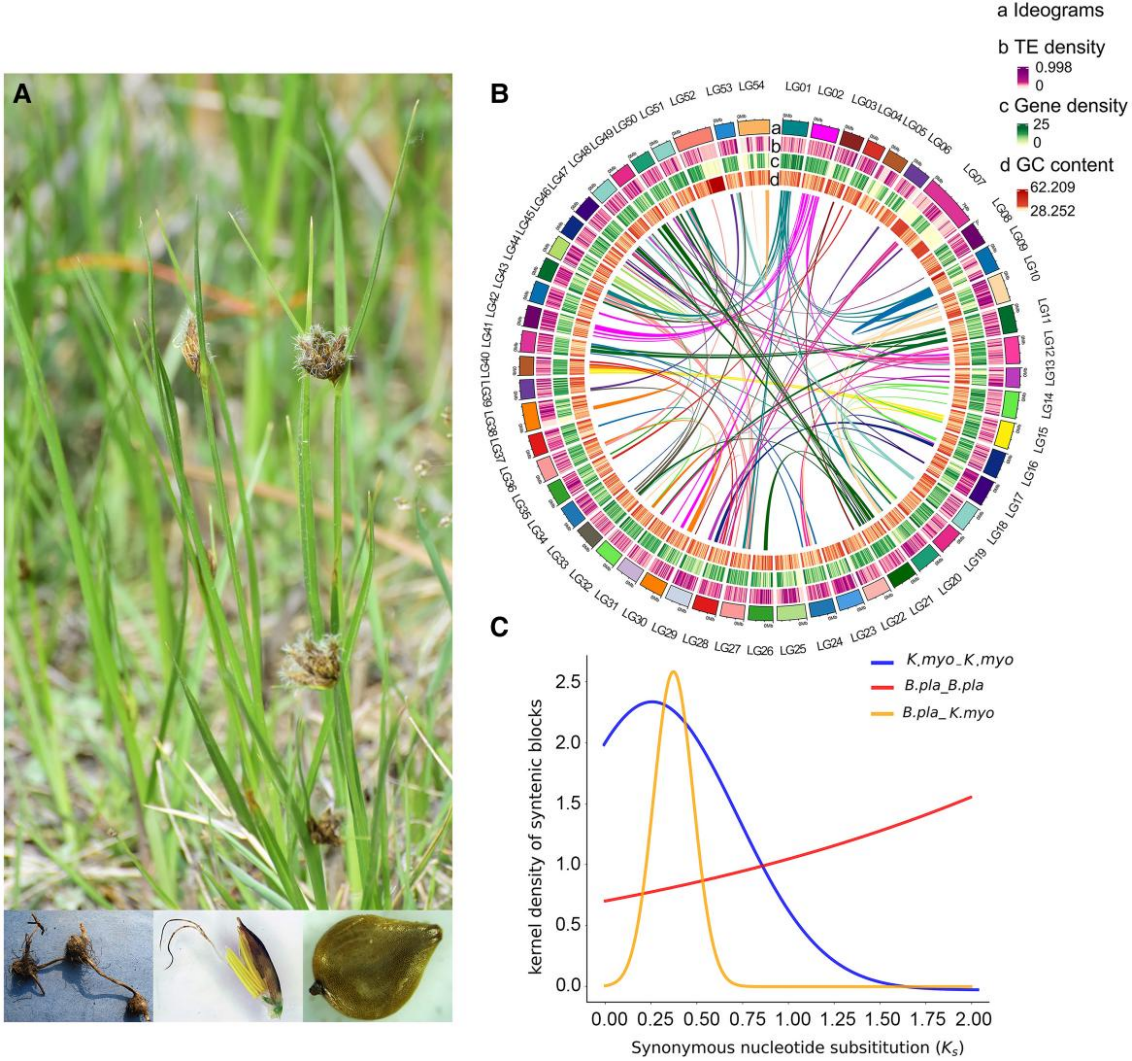
Overview of the *B. planiculmis* genome assembly. A) The sampled individual with corms, a floret, and a seed (inset images below). B) Circos plot showing the distribution of genomic features. The four circular tiers (a–d) represent chromosome ideograms, transposable-element density, gene density, and GC content, respectively. Central lines indicate putative homology among linked sections. The colors of these links are arbitrary and for visual purposes only. C) Gaussian mixture modeling of the *K_s_* distribution of *B. planiculmis* and *Kobresia myosuroides*. No convincing peak was detected in the *B. planiculmis* genome subsequent to its divergence from *K. myosuroides*.

*Bolboschoenus planiculmis* (F.Schmidt) T.V.Egorova (Fig. 1A) is a critical element in wetland ecosystems. The species is a member of the species-rich sedge family (Cyperaceae) and exhibits several unique traits of evolutionary and ecological importance (Ning et al. 2014; An et al. 2018; Ljevnaić-Mašić et al. 2020). By vigorous clonal growth as a result of rhizome elongation and corm formation, *B. planiculmis* and its relatives show strong adaptability to highly disturbed environments, for example, and contribute to the carbon dynamics in global coastal ecosystems (Peng et al. 2022). Although this species is recognized as a notorious weed in paddy fields owing to its strong competitiveness, its starch-rich tubers can provide a food source for wetland birds (An et al. 2018). Moreover, its potential for application in phytoremediation is attracting increasing attention. A recent study has highlighted its propensity for bioaccumulation through adsorption from the rhizosphere, providing an efficient means to remove deleterious ions to prevent their transfer to other trophic levels (Almaamary et al. 2022). However, the available genetic information on *B. planiculmis* is limited, except for the plastid genome (Ning et al. 2022a) and a population genetic analysis based on multilocus genotype data (Píšová et al. 2017). A high-quality reference genome is urgently needed to assist with in-depth scientific research and industrial application of this species.

The evolutionary uniqueness of *Bolboschoenus* plants warrants specific research attention. The Cyperaceae is well known for its exceptional variation in chromosome number (2*n* = 4 to 224), which may have facilitated its extreme diversification and cosmopolitan distribution (Roalson 2008; Hipp et al. 2010; Martín-Bravo et al. 2019). The flexibility in chromosome number may be attributable to the prevalence of holocentric chromosomes in the Cyperaceae. In contrast to monocentric chromosomes with fixed centromeres, holocentricity enables kinetochoric activity along the entire chromosome (Márquez-Corro et al. 2019). This unique trait reduces the chance of abnormalities accompanying chromosome fusion and fission, thus promoting speciation without ploidy change and variation in genome size (i.e. dysploidy) (Escudero et al. 2014). However, karyotypes may evolve differently among the clades of the Cyperaceae. Hipp et al. (2008) suggested that polyploidy is rare in *Carex*, but this may not apply to other Cyperaceae members. A phylogeny-based simulation revealed that the modes of chromosomal evolution were clade specific with different rates and frequencies of fission and fusion events (Márquez-Corro et al. 2019). Nevertheless, no genome assembly has been published for the genus *Bolboschoenus*. A previous study by Jarolímová and Hroudová (1998) has recorded a chromosome count of 54 for *B. planiculmis* at the gametophytic stage, and the Bolboschoeneae lineage tends to host karyotypes with high chromosome number and low average chromosome size (Elliott et al. 2022). Nevertheless, the exact individual chromosome information is still lacking. Thus, the genome assembly presented herein for *B. planiculmis*, together with the associated annotation data, will provide a valuable data source for future evolutionary studies of the Cyperaceae and for practitioners involved in the breeding and engineering of wetland plants.

## Results and Discussion

### Evaluation of the Genome Assembly

We generated 22.79 Gb (∼96×) of PacBio long-read data through circular consensus sequencing (CCS) sequencing, 29.05 Gb (∼122×) of short-read data for Hi-C mapping, 31.09 Gb (∼132×) of Illumina data for the genome profiling, and 13.95 Gb (∼58×) of transcriptomic data to assist in gene modeling (supplementary table S1, Supplementary Material online). The genome profiling revealed that the sampled individual had a small genome of moderate complexity. The genome was approximately 236.00 Mb in size. Repetitive elements accounted for ∼38.18% of the genome. The Guanine-Cytosine (GC) content was estimated to be ∼33.47%, and the inferred heterozygosity was ∼1.76% (see the detailed results of GenomeScope profiling in supplementary figs. S1 and S2, Supplementary Material online).

For genome assembly, we first established a preliminary de novo assembly based on high-quality PacBio CCS long reads (supplementary fig. S3, Supplementary Material online). The final chromosome-level assembly was generated by combining high-quality Hi-C data (supplementary table S3, Supplementary Material online) and the preliminary assembly. Using Hi-C, we were able to detect the association between most contigs and cluster them into pseudomolecules that image the real chromosome identity. In our case, 54 pseudochromosomes were constructed with an anchored rate of 93.34%, meaning 93.34% of the total base were mapped into these pseudochromosomes; thus, the preliminary assembly was polished to chromosome level (supplementary fig. S4, Supplementary Material online; Table 1). The final genome assembly of *B. planiculmis* comprised a total of 238.01 Mb (contig N50 value of 3.61 Mb, GC content 35.30%). This final assembly included both the well-anchored and nonanchored bases. We constructed a chromosome ideogram that represented the collinearity relations, GC content, transposable elements (TEs), and gene density (Fig. 1B). The genome size was consistent with the results of the genome profiling (supplementary fig. S1, Supplementary Material online) and flow cytometry (supplementary fig. S2 and table S2, Supplementary Material online).

**Table 1:** Statistics for the *B. planiculmis* genome assembly and BUSCO scores

Type	Statistics
*Sequence*	
Assembly size (bp)	238,008,122
GC content (%)	35.30
Number of scaffolds	148
Longest scaffold (bp)	9,066,051
Scaffold N50 size (bp)	4,037,645
Number of contigs	168
Longest contig (bp)	5,698,134
Contig N50 size (bp)	3,612,616
*Pseudochromosome*	
Number	54
Anchored rate (%)	93.34
Size range (M)	3.16 ∼ 9.41
*BUSCO score*	
Complete BUSCOs (%)	95.48
Complete and single-copy BUSCOs (%)	93.99
Complete and duplicated BUSCOs (%)	1.49
Fragmented BUSCOs (%)	0.31
Missing BUSCOs (%)	4.21
Total groups searched	1,614

“Anchored rate” refers to the proportion of bases that are well mapped into pseudochromosomes. Those unmapped bases are also included in the final assembly. “Size range” delimits the minimum and maximum size of pseudochromosomes.

The high quality of the assembly was attested by the following evidences: (i) the mapping-back rate and average depth were high for both long reads (98.68%, 86×) and short reads (96.04%, 107×) (details provided in supplementary table S4, Supplementary Material online); and (ii) the complete BUSCO score of 95.48% (Table 1) was comparable with those recently reported for four cyperid genomes (Planta et al. 2022). (iii) Of all the 54 chromosomes, 48 (∼88.89% of the total number) showed signals of telomeres, among which 32 chromosomes exhibited telomeres at both ends (supplementary fig. S5, Supplementary Material online). Notably, the present assembly showed that *B. planiculmis* has a haploid chromosome number of *n* = 54. This value was consistent with the results of a previous cytological study (Jarolímová and Hroudová 1998) and was considerably higher than those reported for all published cyperid genomes (Can et al. 2020; Hofstatter et al. 2022; Ning et al. 2022b; Planta et al. 2022). The average pseudochromosome size was small (∼4.1 Mb), with 43 (∼80%) pseudochromosomes corresponding to only one contig (supplementary table S5, Supplementary Material online). Moreover, the proportion of duplicated BUSCO scores was extremely low (1.49%; Table 1). Compared with the genome of *Rhynchospora pubera*, which has a duplication percentage of 95.29% attributed to whole-genome duplication (WGD) events (Hofstatter et al. 2022), the genome of *B. planiculmis* is unlikely to have undergone lineage-specific duplication bursts. Instead, dysploidy evolution, which leads to variation in chromosome number but limited change in genome size, may better explain the karyotype evolution of this species.

### Synteny and Karyotype

To evaluate support for the inferred absence of recent WGD events in *B. planiculmis*, we investigated the intragenomic synteny and synonymous substitution rate (*K_s_*) distribution of the assembled genome. Neither the synteny block (segments with a high confidence of synteny) plot (supplementary fig. S6A, Supplementary Material online) nor the original unfiltered synteny dot plot (supplementary fig. S6B, Supplementary Material online) showed convincing evidence for lineage-specific WGD events. Regarding *K_s_* distribution modeling, we used the genome assembly of *Kobresia myosuroides* as a reference. The results provided no indication of an intensive burst of synonymous substitutions in the *B. planiculmis* genome after its divergence from *K. myosuroides* (Fig. 1C). Gaussian mixture modeling failed to fit a unimodal distribution (supplementary fig. S7, Supplementary Material online). Combined with the extremely low percentage of duplicated BUSCO scores, it was concluded that no lineage-specific WGD event was likely to have contributed to the evolution of the *B. planiculmis* genome. Previous models have shown that the Fuireneae–Abildgaardieae–Eleocharideae–Cypereae clade (including *Bolboschoenus*) in the Cyperaceae experiences a 3-fold increase in diversification rate and the evolution of a majority of this clade is dominated by high rates of fusion and fission events (Escudero and Hipp 2013; Márquez-Corro et al. 2019). The present genome assembly supports the duplication-limited and dysploidy-prone evolutionary mode.

### Genome Annotation and Gene Features

Approximately 37.04% (∼88.17 Mb) of the *B. planiculmis* genome comprised repetitive sequences. TEs constituted 21.81% of the genome and tandem repeats accounted for 15.23% of the genome. Detailed information for these two categories is shown in supplementary tables S6 and S7, Supplementary Material online. Among the various components of TEs, long terminal repeat retrotransposons (LTR-RTs) comprised ∼9.62% of the genome. It is well documented that variation in activities of LTR-RTs is largely responsible for genome size evolution (Zhao and Ma 2013). Thus, the relation between the low proportion of LTR-RTs and the small genome of *B. planiculmis* merits further investigation. In addition, a consensus of low proportions of LTR-RTs (6.1% to 15.4% of the genome) is evident in other genome assemblies in the Cyperaceae (Planta et al. 2022). Previous studies have shown that LTR-RTs and satellite repeats tend to be enriched in centromeric regions (Zhao and Ma 2013), and an edging study has highlighted the evolutionary significance of repeat-based holocentromeres in the genomes of three beak-sedges (*Rhynchospora* spp.) (Hofstatter et al. 2022). Thus, we established a thorough search for centromeres using quarTeT (Lin et al 2023). The results indicated that not all the chromosomes in *B. planiculmis* have multiple centromeres. Specifically, ten chromosomes showed no traces of centromere. Fifteen chromosomes were monocentric. The rest 29 chromosomes tended to have multiple centromeres (details in supplementary fig. S5, Supplementary Material online). We also noticed that the longest chromosome (Chr LG07) hosted the highest number of centromeres (seven candidates). Our results suggest that the distribution of centromeres may be heterogeneous among chromosomes. Thus, the importance of repeat elements in clubrush needs further investigation.

After masking the repeats, we established a gene model that integrated three methods (based on ab initio, homology, and transcriptome sequencing data, respectively). In total, 18,760 protein-coding genes were predicted in the *B. planiculmis* genome (supplementary table S8, Supplementary Material online). Approximately 82.79% of these predicted genes were supported by all three methods. The overall BUSCO score for the gene prediction was 94.18% (complete and single-copy 92.26% and complete and duplicated 1.92%). Further analysis revealed that 98.94% of the predicted genes were annotated in the public databases (EggNOG, NR, Swiss-Prot, KEGG, Pfam, KOG, TrEMBL, and GO; details are provided in supplementary table S8, Supplementary Material online). In addition, a noncoding RNA library was constructed, comprising 3,689 rRNAs, 487 tRNAs, 100 miRNAs, 50 snRNAs, and 21 snoRNAs. Furthermore, 22 pseudogenes were detected with a total length of 39,709 bp.

## Materials and Methods

### Collection and Preparation of Plant Materials

Healthy individuals of *B. planiculmis* were gathered from the Changgou Wetland (39°35′13″N, 115°53′32″E). We carefully chose healthy, clean leaves for DNA extraction and genomic sequencing. All samples were handled with care to prevent contamination by external pollutants and stored at −80 °C. A voucher specimen is housed in the herbarium of the National Natural History Museum of China (ID: BJM0272524, available upon reasonable request from the corresponding author).

### Genome Sequencing

Genomic DNA was extracted using the cetyltrimethylammonium bromide method. Following a standard protocol, we generated a 15-kb DNA SMRTbell library for CCS sequencing. For Hi-C library construction, we followed a previously published protocol involving *Hin*dIII enzymatic digestion (Xie et al. 2015). An Illumina NovaSeq 6000 platform was used for Hi-C library sequencing.

### Transcriptome Sequencing

We collected fresh samples from five tissues (corm, spikelet, stem, root, and leaf) for RNA sequencing. Paired-end libraries were generated using the conventional mRNA-seq prep kit following the manufacturer's instructions. The insertion size was approximately 350 bp on average. An Illumina NovaSeq 6000 platform was used to sequence the RNA libraries.

### Genome Profiling and Draft Assembly

Sequences generated from the pair-end libraries were filtered to survey the genome. Inspection was performed using Genome Scope (v. 2.0) (Ranallo-Benavidez et al. 2020) and Jellyfish (v. 2.1.4) (Marçais and Kingsford 2011). A 19-nt *k*-mer distribution model was ultimately selected. In addition, flow cytometry experiments were conducted to validate the estimate of the genome size.

Clean data for the PacBio long reads were assembled using HIFIASM (v. 0.14) (Cheng et al. 2021). The preliminary contigs were then adjusted using Pilon (v. 1.24) (Walker et al. 2014). We utilized the Burrows–Wheeler Aligner (BWA; v. 0.7.17) and SAMtools (v. 1.13) to obtain alignments and for file format conversion (Li and Durbin 2009). Assembly integrity and completeness were assessed using BUSCO (v. 3.0) (Waterhouse et al. 2017). The embryophyta_odb10 database was chosen as the reference.

### Finalizing the Genome Assembly Using Hi-C Technology

The clean Hi-C data were truncated according to the ascertained junction sites and then aligned with the draft assembly using BWA. For subsequent analysis, only read pairs that were uniquely aligned with mapping quality > 20 were retained. HiC-Pro (v. 2.8.1) (Servant et al. 2015) was used to filter out invalid read pairs.

Initially, we divided the preliminary scaffolds into partitions of ∼50 kb with error correction. The validated Hi-C data were then aligned onto these partitions using BWA, and only singularly aligned data were retained and further processed with LACHESIS (Burton et al. 2013). We carefully checked and corrected any erroneous placements or orientations that exhibited obvious discrete chromatin interaction patterns.

### Gene Prediction and Annotation

To identify high-quality protein-coding genes, we used the MAKER pipeline (Cantarel et al. 2007). The de novo gene models were generated by implementing two ab initio gene prediction software tools (Augustus and SNAP). Homology detection was executed using four monocotyledonous species as references (*Oryza sativa*, *Triticum aestivum*, *Panicum virgatum*, and *Carex littledalei*). For transcript-based prediction, the RNA-sequencing data were used to generate unigenes for gene predictions. Finally, the outcomes of the three methods were reconciled using EVM (v. 1.1.1) (Haas et al. 2008).

To annotate the functions of genes in the *B. planiculmis* genome, we conducted a BLASTP search (details are provided in supplementary table S8, Supplementary Material online) with an *E*-value threshold of 1.0 × 10^−5^. We used tRNAscan-SE (v. 1.3.1) (Lowe and Eddy 1997) to detect tRNA with eukaryote parameters. We used Barrnap (v. 0.9) (Loman 2017) to identify rRNA genes. The miRNA, snoRNA, and snRNA genes were determined using INFERNAL1.1 (Nawrocki and Eddy 2013) with reference to the Rfam (release 12.0) database; detailed procedures could be found in the manual of IINFERNAL (http://eddylab.org/infernal/).

### Detection of Repetitive Elements

We first built a de novo repeat library using RepeatModeler2 (v. 2.0.1) (Flynn et al. 2020) and then identified and retrieved high-quality intact LTRs using LTR_retriever (v. 2.8) (Ou and Jiang 2017). After removing redundant repeats, the final species-specific TE library was obtained through a combination of database mapping and correction with RepeatMasker (v. 4.10) (Tarailo-Graovac and Chen 2009). In addition, we used TRF (Benson 1999) and MISA (v. 2.1) (Beier et al. 2017) to identify tandem repeats and enhance the dimensions of the repetitive element library. We screened the genome for potential centromeres and telomeres using quarTeT (http://www.atcgn.com:8080/quarTeT/home.html) (Lin et al 2023) and visualized the results using RIdeogram (v. 0.2.2) (Hao et al 2020).

### Detection of Intragenome Synteny and Potential Duplication Bursts

The WGDI toolkit (Sun et al. 2022) was used to ascertain intragenomic synteny and potential duplication bursts. WGDI constructs a hierarchical algorithm to improve the sensitivity and accuracy of collinearity detection. The functions “-d”, “-icl”, “-ks”, “-bi”, “-bk”, “-kp”, and “-kf” were invoked following the toolkit instructions (https://wgdi.readthedocs.io/en/latest/Introduction.html). Finally, an ideogram of pseudochromosomes was generated to intuitively represent the multidimensional genomic information for *B. planiculmis*.

## Supplementary Material

evae039_Supplementary_Data

## Data Availability

The reported genome assembly is archived in NCBI under accession number PRJNA937020. Biosample information could be accessed at SAMN33386850. The read data involved in this research are deposited at Sequence Read Archive (SRA) with accession numbers SRR23592699 (Illumina short reads) and SRR23592700 (PacBio long reads). The related annotation files could be found at https://figshare.com/projects/genome_annotations_files_for_Bolboschoenus_planiculmis/162637, including coding sequences, protein sequences, and functional annotation in gff3 format.
