# Supplementary material for: Chromosome-Scale Genome Assembly for Clubrush (Bolboschoenus planiculmis) Indicates a Karyotype with High Chromosome Number and Heterogeneous Centromere Distribution
Source: Genome Biol Evol. 2024 Mar 6;16(3):evae039. doi: 10.1093/gbe/evae039 (PMC10959549; doi:10.1093/gbe/evae039)
Supplement: evae039_Supplementary_Data [file evae039_supplementary_data.docx]

|  |
| --- |

**Supplementary materials for “Chromosome-scale genome assembly for clubrush (*Bolboschoenus planiculmis*) indicates a karyotype with high chromosome number and heterogeneous centromere distribution”**

NING, Yu^1,3^; LI, Yang^2^;LIN, Hai Yan^4^; KANG, En Ze^1,3^;ZHAO, Yu Xin^5^; DONG, Shu Bin^5^; LI, Yong^1,3^; XIA, Xiao Fei^6^; WANG, Yi Fei^1,3^

*1: Wetland Research Center, Institute of Ecological Conservation and Restoration, Chinese Academy of Forestry*

*2:* *Huzhou University, Huzhou, China;*

*3: Sichuan Zoige Wetland Ecosystem Research Station, Tibetan Autonomous Prefecture of Aba, China*

*4: Institute of Information Technology, Chongqing Academy of Forestry Sciences*

*5: College of Biological Sciences and Technology, Beijing Forestry University*

*6: National Natural History Museum of China*

^*^Corresponding author. Email: [wangyifei.af@126.com](mailto:wangyifei.af@126.com)

Address: 2#, Xiangshan Road, Haidian District, Beijing, China.

Phone &Fax: +86 01062824182


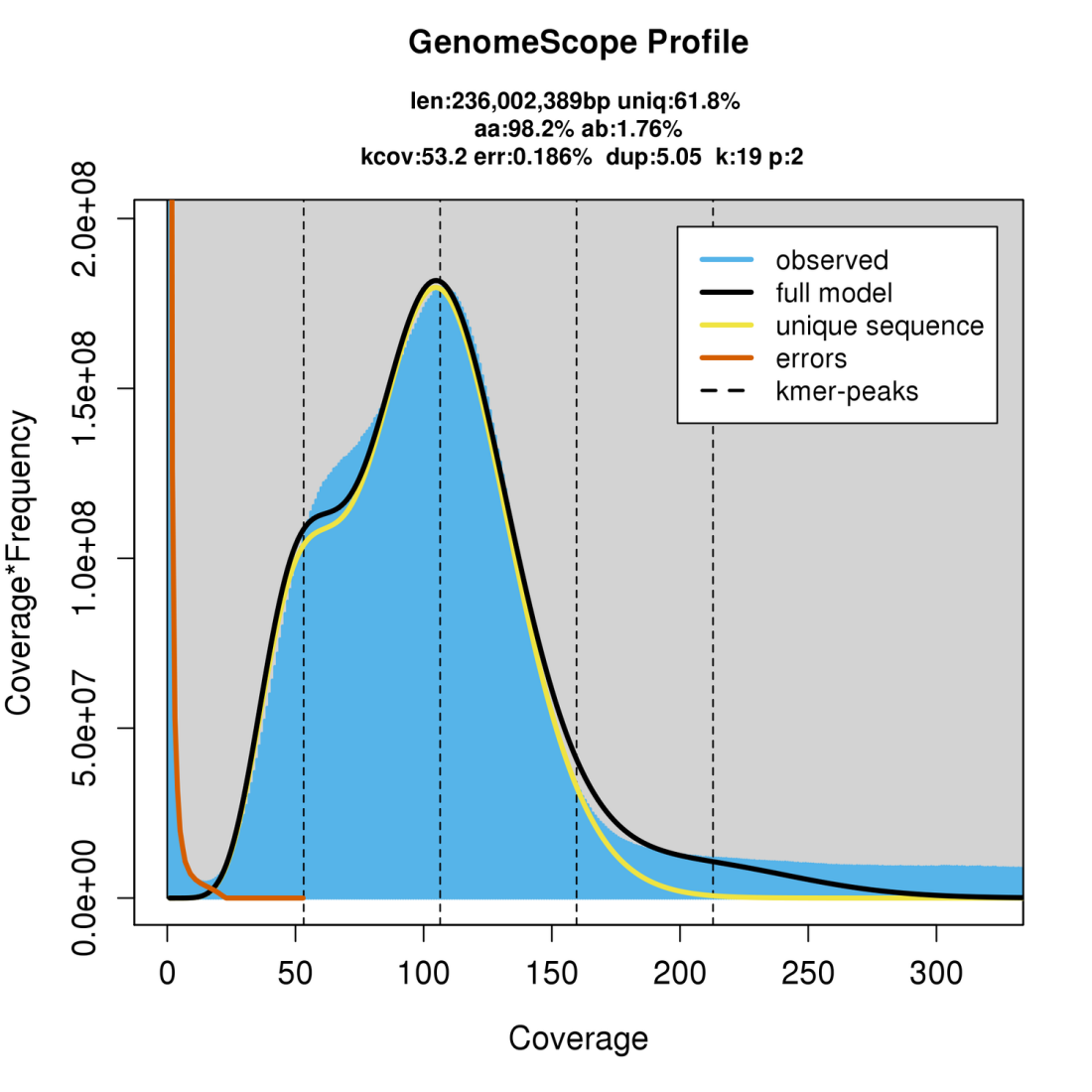


**Supplementary Figure S1. The *K*-mer analysis used to evaluate** ***Bolboschoenus planiculmis* genome.** The frequency distributions of 19-mers were shown.The genome size of *B.planiculmis* was estimated to be ~ 236 Mb, with approximate repetitive content of 38.18%,GC content of 33.47% and heterozygosity of 1.76%. The putative ploidy level is 2X.

| 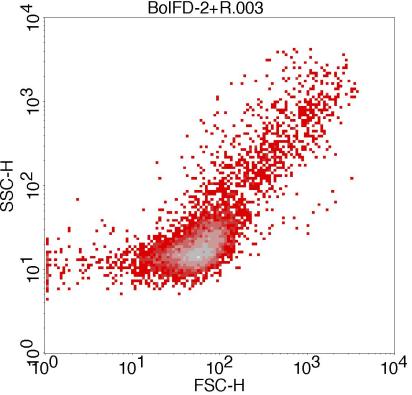 | 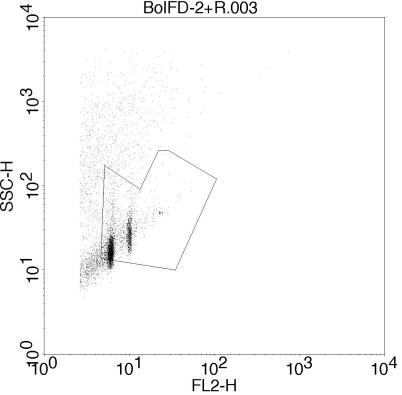 | 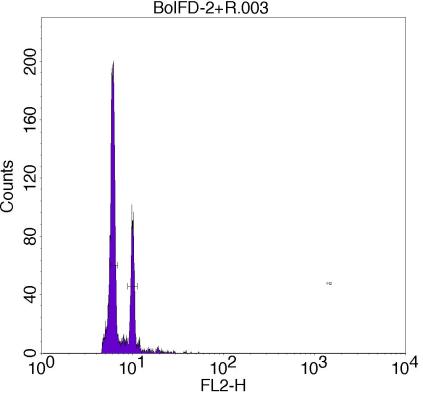 |
| --- | --- | --- |
| 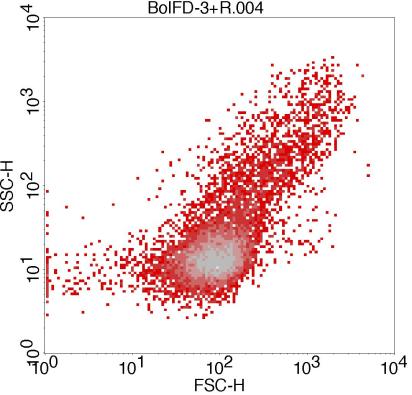 | 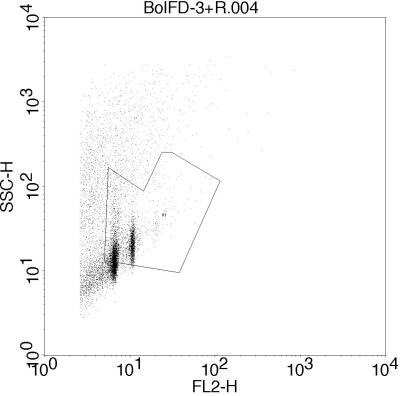 | 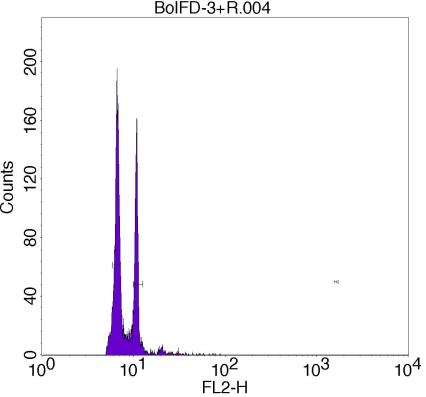 |
| 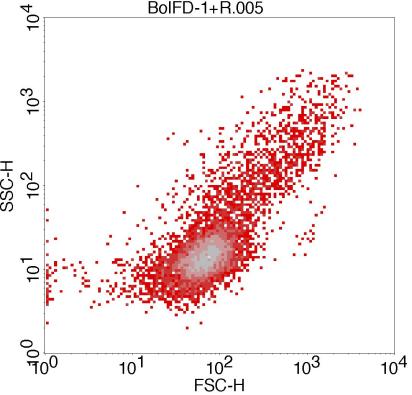 | 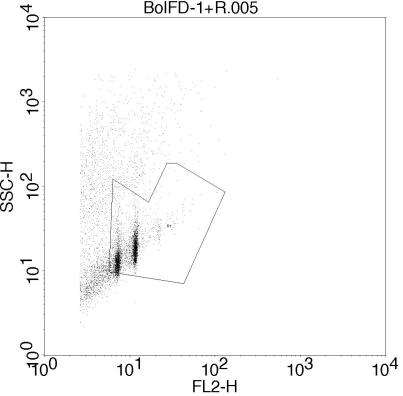 | 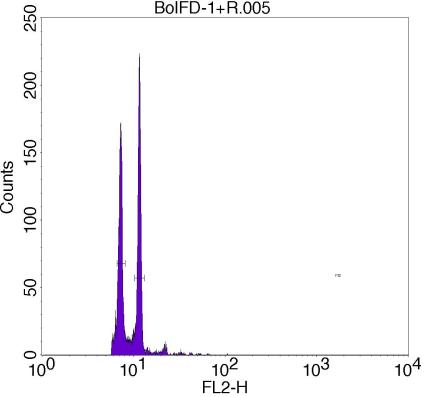 |
| 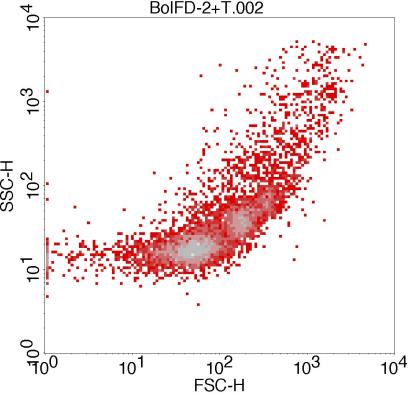 | 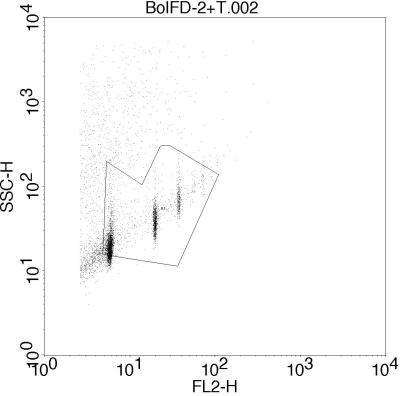 | 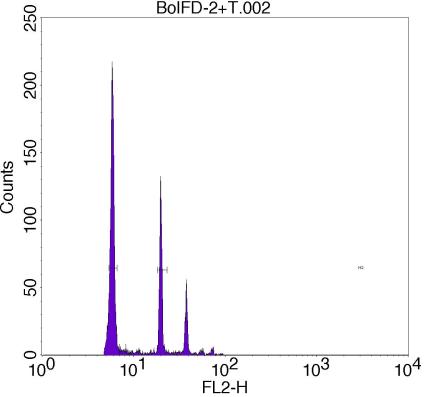 |

**Supplementary Figure S2. The result of flow cytometry of *Bolboschoenus planiculmis* using rice genome (R.003-R.005 ) and tomato genome (T.002 ) as reference.** Detailed value are shown in supplementary table 2.


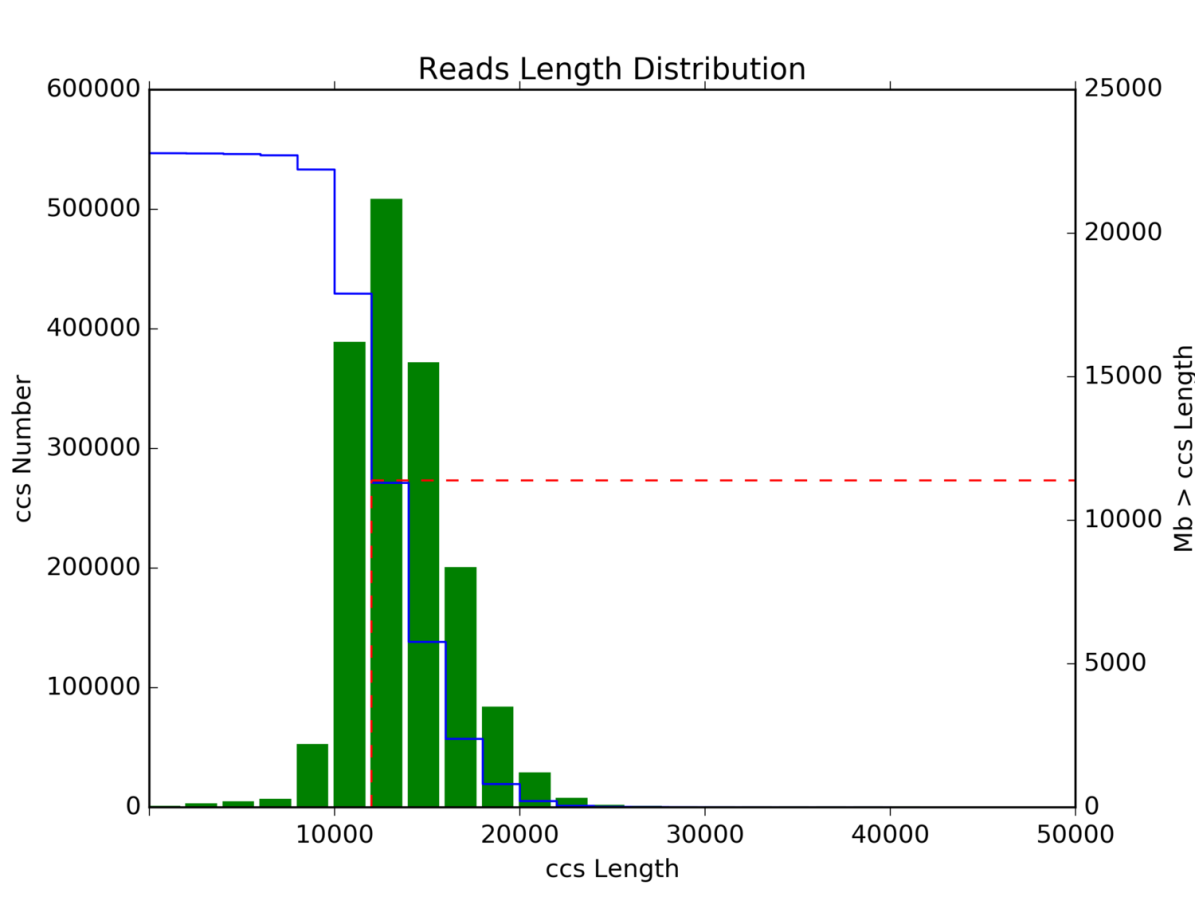


**Supplementary Figure S3. The distribution of CCS reads length.** The bottom X-axis represents the gradients of CCS reads length. The left Y-axis shows the number of CCS reads. The blue curve denotes the accumulated base volume which are assigned to reads longer than the corresponding value on the right Y-axis. The red-dotted line exhibits the N50 value of CCS reads.

**
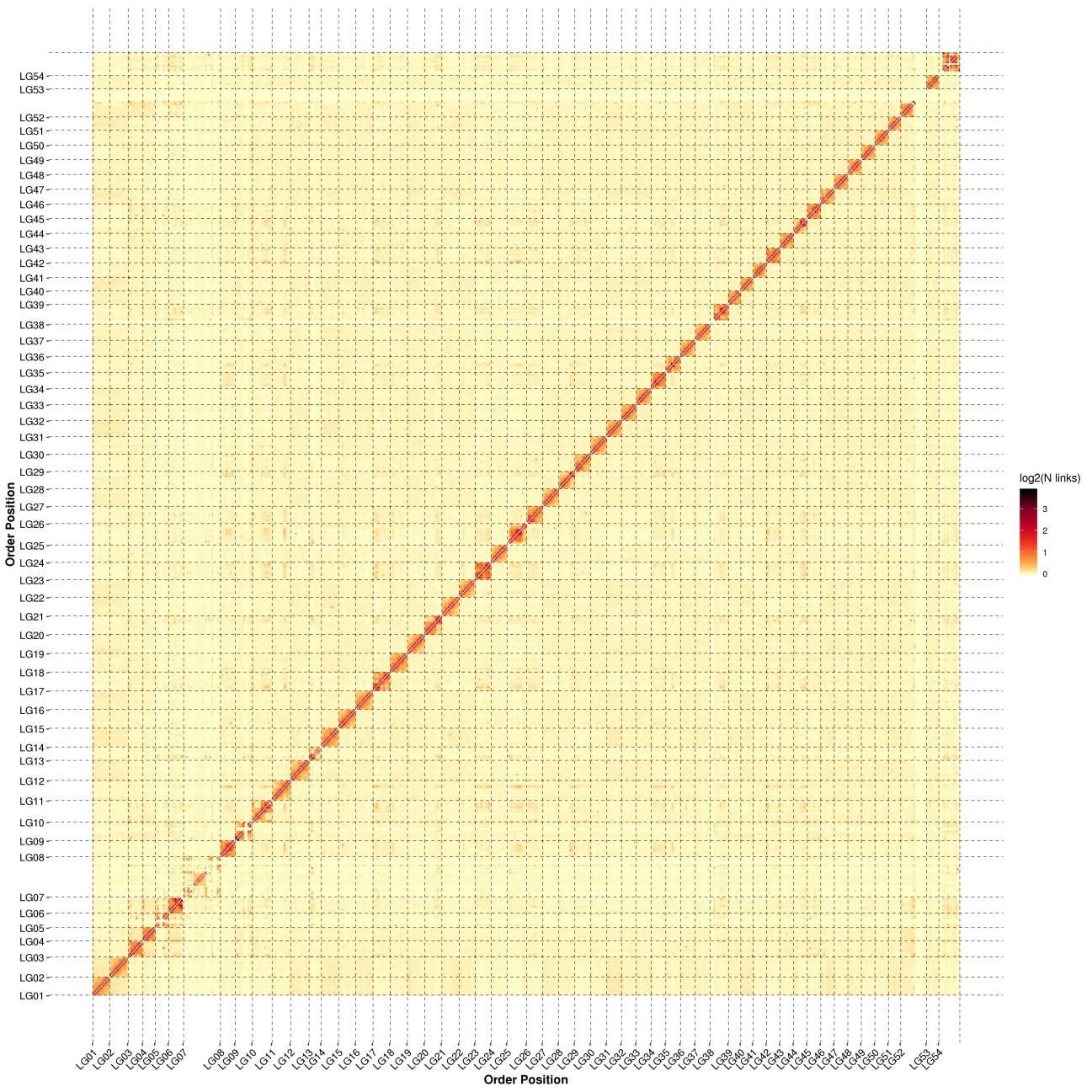
**

**Supplementary Figure S4. Heatmap of Hi-C interactions of the *B.planiculmis* pseudochromosomes** The resolution is 300 kb. Colour gradients from yellow to red indicate the frequencies of Hi-C links alter from low to high.

| 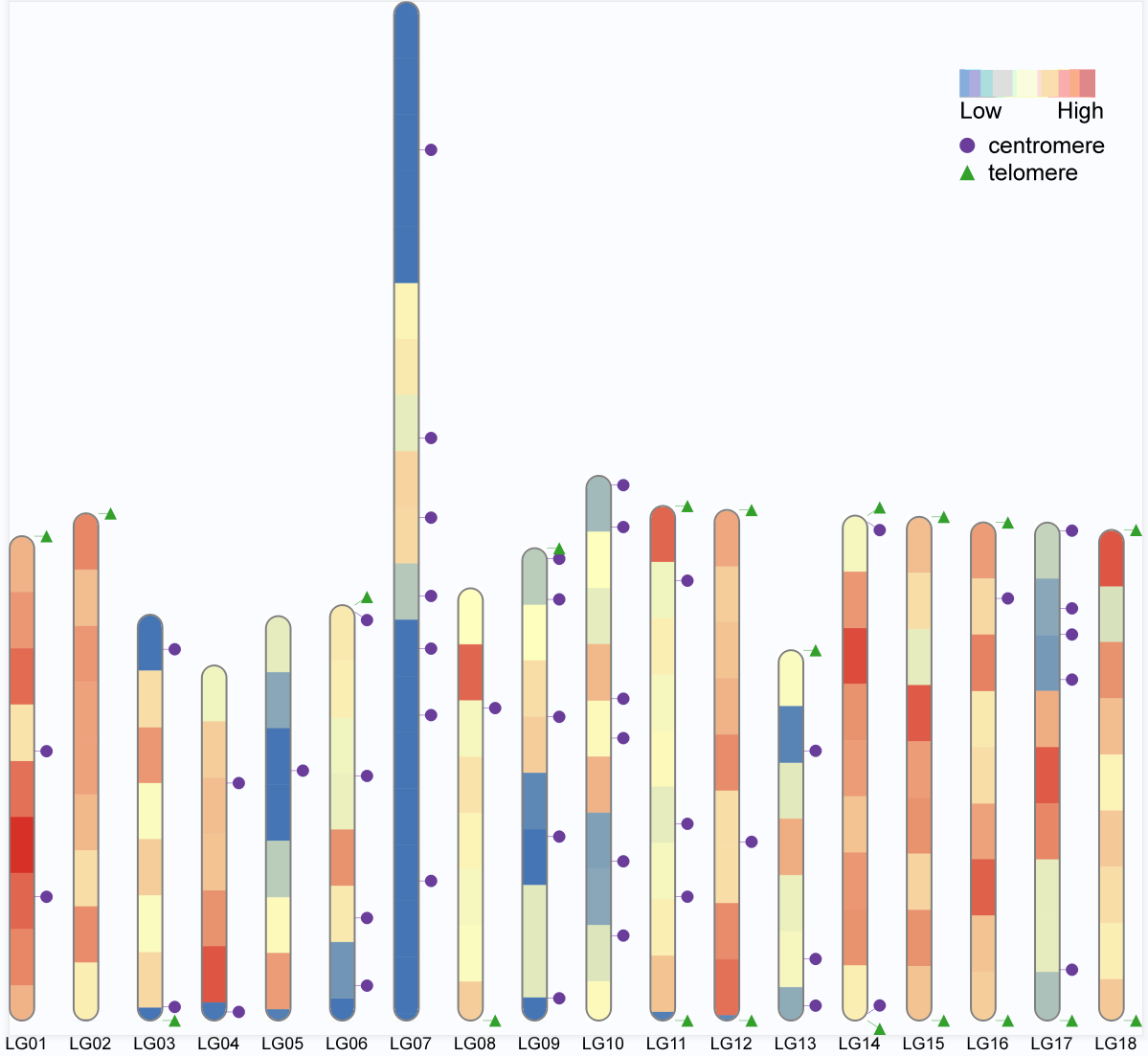 |
| --- |
| 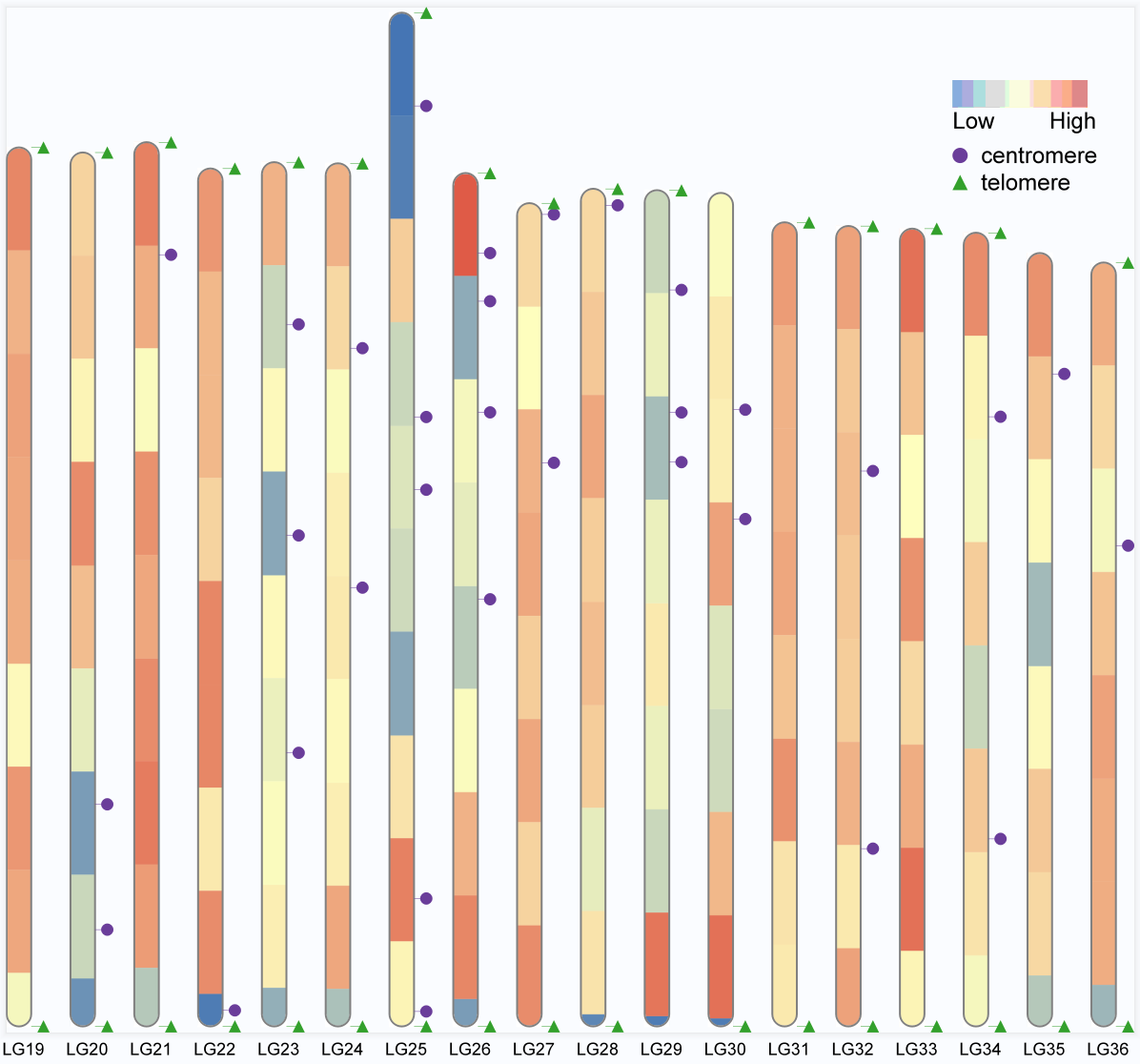  **Supplementary Figure S5. Illustration of telomeres and centromeres detected in the present genome assembly.** Each chromosome is filled with color scalling to the gene density at 500kb intervals. The positions of detected telomeres and centromeres are marked with different symbols. |
| 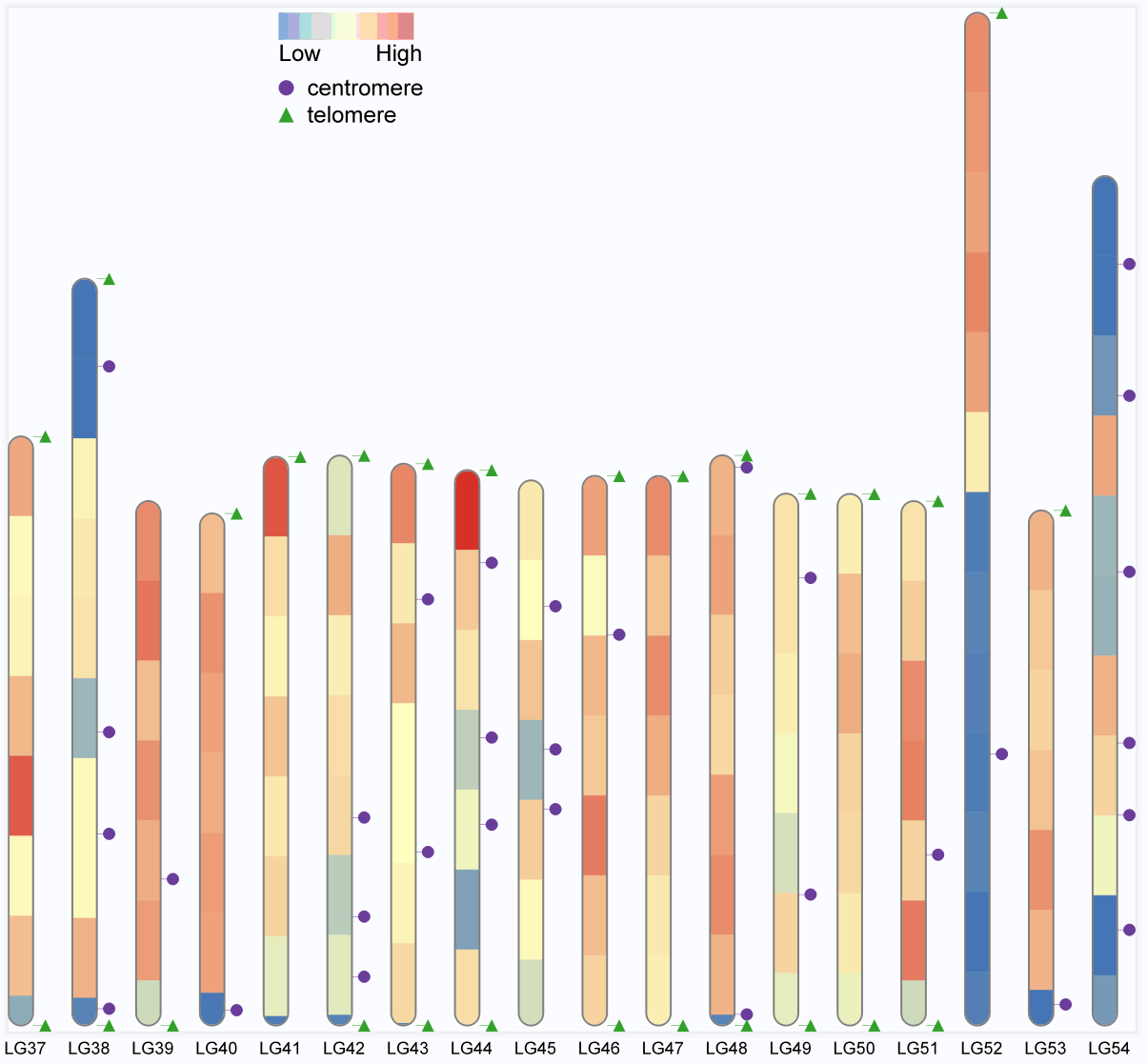 |

| **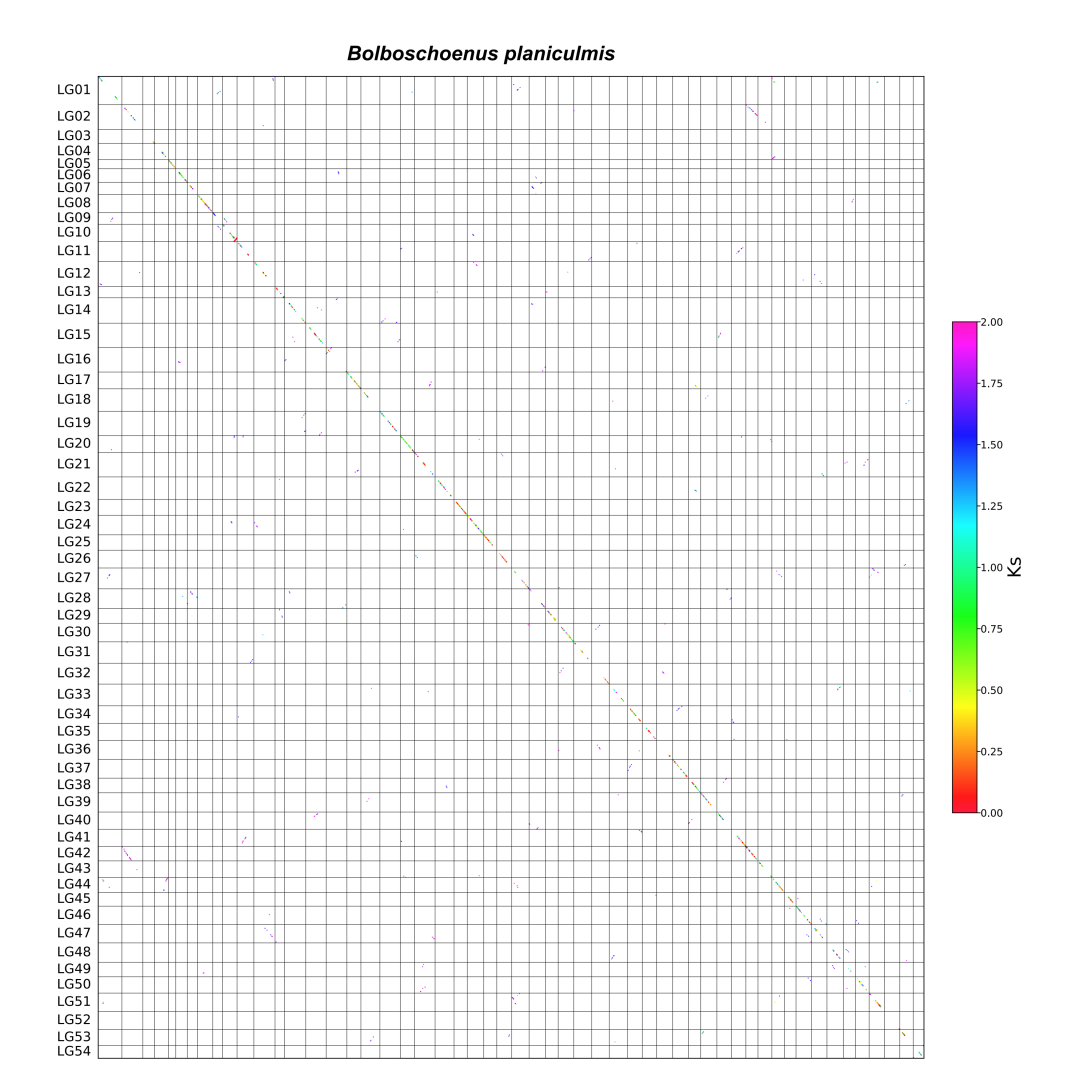**  **a** |
| --- |
| 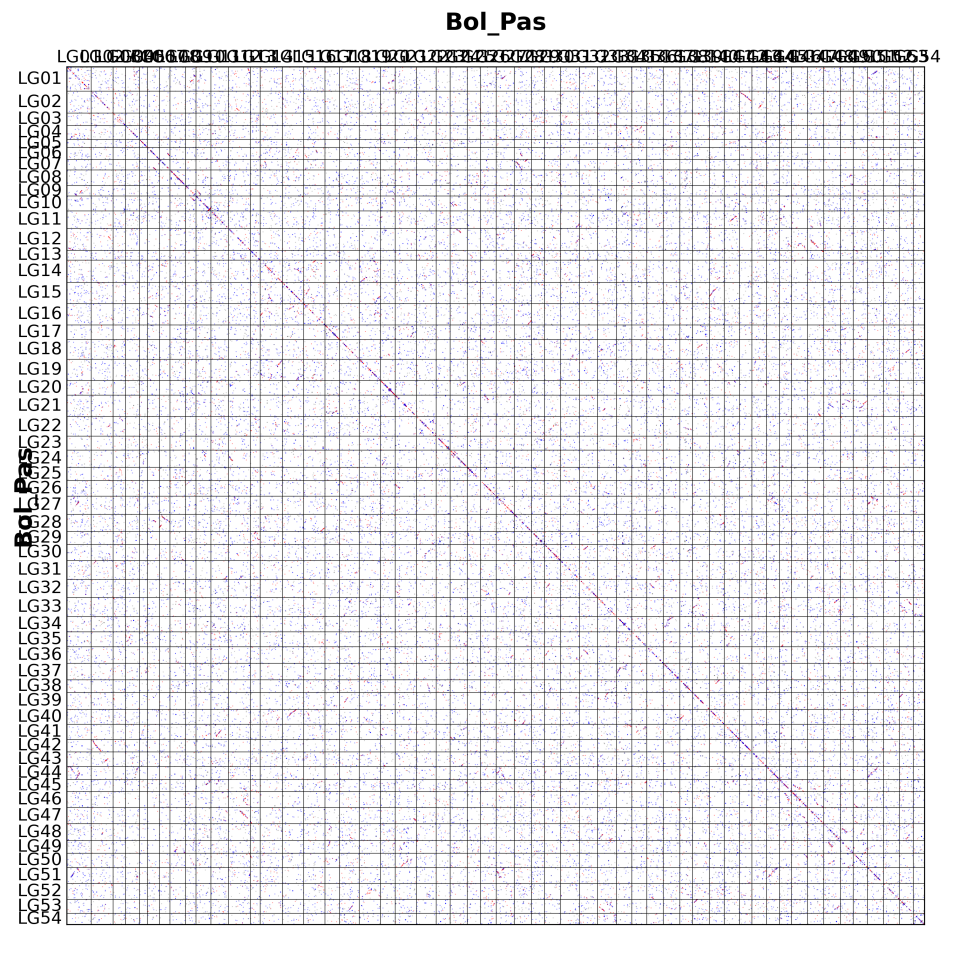  **Supplementary Figure S6. Filtered collinearity dot plot of gene pairs of *B. planiculmis* (a) and original unfiltered dot plot (b)** Ks values are indicated by color gradients. No convincing segments of synteny blocks have been detected.  **b** |

| **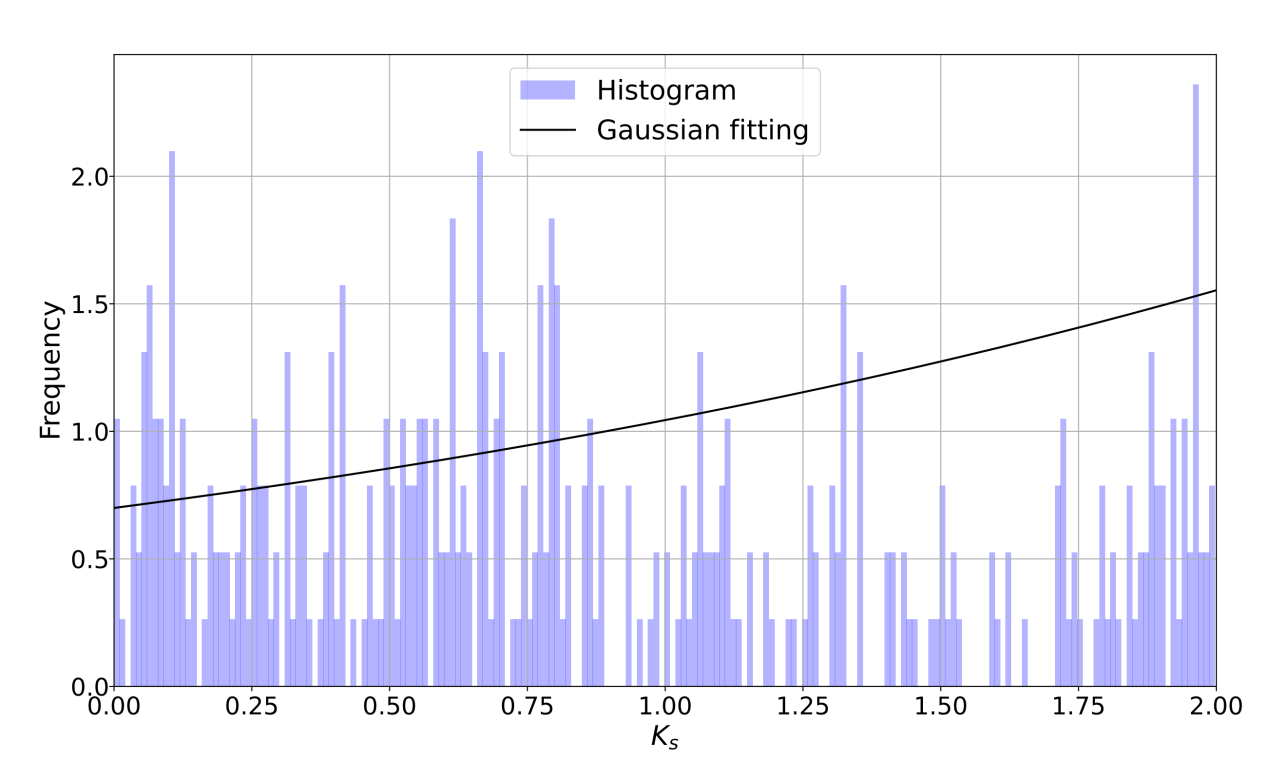**  **a**  R^2^=0.333 |
| --- |
| **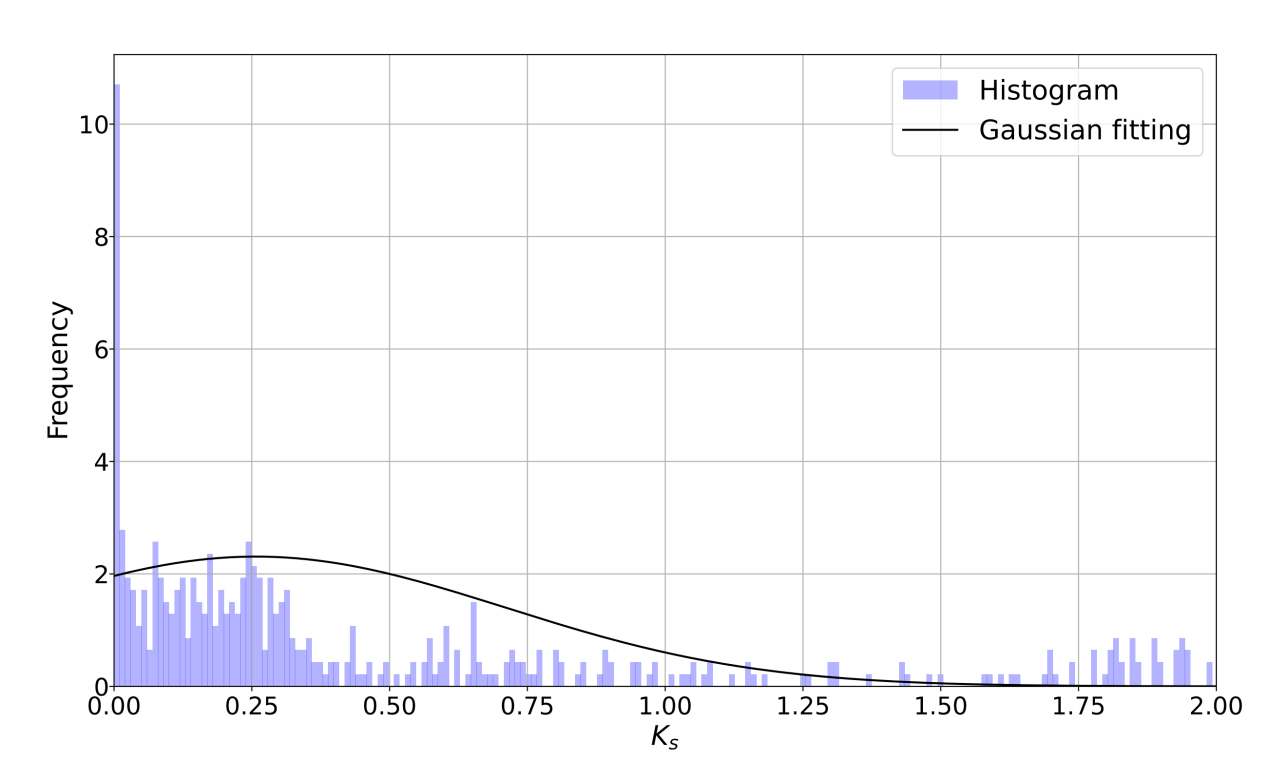**  **b**  R^2^=0.561 |

**Supplementary Figure S7. Modeling of Ks distribution of *B. planiculmis* (a) and *K. myosuroides* (b).** None of the fitting get sufficient support, indicating little confidence of recent WGD event.

**Supplementary Table S1. Summaries of data volume and quality**

| **Application** | **Library type** | **Platform** | **Total Data (Gb)** | **Reads Number** | **Q30(%)** |
| --- | --- | --- | --- | --- | --- |
| preliminary assemble | Pacbio | Sequel | 22.79 | 1,654,129 | --- |
| Hi-C anchoring | Illumina | NovaSeq 6000 | 29.05 | 97,046,445 | 93.18 |
| survey | Illumina | NovaSeq 6000 | 31.09 | 208,139,332 | 92.46 |
| transcriptome | Illumina | NovaSeq 6000 | 13.95 | 46,802,358 | 94.56 |

**Supplementary Table S2.** **Results of flow cytometry of *Bolboschoenus planiculmis***

| **SampleID** | **Reference C-value(Mb)** | **Reference fluorescence density** | **Sample fluorescence density** | **Ratio** | **Estimated C-value(Mb)** |
| --- | --- | --- | --- | --- | --- |
| BolFD-1 | 430 | 11.37 | 6.99 | 0.615 | 258.21 |
| BolFD-2 | 430 | 9.75 | 5.94 | 0.609 | 255.88 |
| BolFD-2 | 880 | 19.49 | 5.75 | 0.295 | 259.62 |
| BolFD-3 | 430 | 10.56 | 6.44 | 0.610 | 256.14 |
|  |  |  |  |  |  |
| *Average* |  |  |  |  | *257.46* |

**Supplementary Table S3 Assessment of Hi-C library quality based on ratio of read pairs with valid interaction**

| **Type** | **Number** | **Ratio(%)** |
| --- | --- | --- |
| ***Valid Interaction Pairs*** | 35542908 | 46.8 |
| ***Invalid Interaction Pairs*** |  |  |
| Dangling End Pairs | 27288147 | 35.93 |
| Re-ligation Pairs | 3151153 | 4.15 |
| Self-cycle Pairs | 940468 | 1.24 |
| Dumped Pairs | 9022718 | 11.88 |

**Supplementary Table S4** **Assessment of assembly quality through mapping back rates and coverage score of both kinds of reads**

|  |  | **Illumina reads** | **Pacbio reads** |
| --- | --- | --- | --- |
| mapping | Total number of reads | 208,139,332 | 1,654,129 |
|  | number of mapped reads | 199,892,059 | 1,632,350 |
|  | Mapping rate (%) | 96.04 | 98.68 |
|  |  |  |  |
| coverage & depth | Coverage 1×(%) | 99.90 | 99.99 |
|  | Coverage at least 5× (%) | 99.63 | 99.67 |
|  | Coverage at least 10× (%) | 99.31 | 98.34 |
|  | Coverage at least 20× (%) | 98.31 | 93.39 |
|  | Average depth | 107 | 86 |

**Supplementary Table S5** **Summary of *B. planiculmis* pseudochromosomes**

| **ChromosomeID** | **Length (bp)** | **Number of contigs** | **Number of genes** |
| --- | --- | --- | --- |
| LG01 | 4,311,779 | 1 | 535 |
| LG02 | 4,514,732 | 1 | 472 |
| LG03 | 3,635,733 | 2 | 267 |
| LG04 | 3,160,892 | 1 | 309 |
| LG05 | 3,600,000 | 1 | 169 |
| LG06 | 3,697,722 | 2 | 261 |
| LG07 | 9,405,294 | 4 | 229 |
| LG08 | 3,847,307 | 3 | 343 |
| LG09 | 4,204,190 | 1 | 222 |
| LG10 | 4,848,474 | 5 | 328 |
| LG11 | 4,580,628 | 1 | 379 |
| LG12 | 4,545,057 | 1 | 477 |
| LG13 | 3,296,652 | 1 | 211 |
| LG14 | 4,494,637 | 1 | 483 |
| LG15 | 4,483,816 | 1 | 462 |
| LG16 | 4,433,955 | 2 | 466 |
| LG17 | 4,432,314 | 1 | 318 |
| LG18 | 4,367,199 | 1 | 428 |
| LG19 | 4,257,965 | 1 | 460 |
| LG20 | 4,254,704 | 3 | 322 |
| LG21 | 4,283,481 | 1 | 463 |
| LG22 | 4,155,960 | 1 | 428 |
| LG23 | 4,186,659 | 1 | 299 |
| LG24 | 4,181,024 | 1 | 372 |
| LG25 | 4,910,726 | 2 | 294 |
| LG26 | 4,133,838 | 2 | 336 |
| LG27 | 3,988,139 | 1 | 395 |
| LG28 | 4,057,730 | 1 | 372 |
| LG29 | 4,050,566 | 3 | 283 |
| LG30 | 4,037,645 | 1 | 351 |
| LG31 | 3,895,281 | 1 | 406 |
| LG32 | 3,877,454 | 1 | 397 |
| LG33 | 3,864,603 | 1 | 411 |
| LG34 | 3,844,858 | 1 | 336 |
| LG35 | 3,746,341 | 1 | 320 |
| LG36 | 3,700,540 | 1 | 362 |
| LG37 | 3,685,084 | 1 | 356 |
| LG38 | 5,761,206 | 2 | 277 |
| LG39 | 3,404,244 | 3 | 367 |
| LG40 | 3,203,498 | 1 | 328 |
| LG41 | 3,557,702 | 1 | 325 |
| LG42 | 3,565,639 | 1 | 274 |
| LG43 | 3,514,482 | 1 | 314 |
| LG44 | 3,474,388 | 1 | 283 |
| LG45 | 3,446,069 | 2 | 262 |
| LG46 | 3,438,538 | 1 | 349 |
| LG47 | 3,437,572 | 1 | 349 |
| LG48 | 3,567,085 | 1 | 370 |
| LG49 | 3,327,389 | 1 | 272 |
| LG50 | 3,325,647 | 1 | 311 |
| LG51 | 3,280,547 | 1 | 346 |
| LG52 | 6,335,059 | 3 | 345 |
| LG53 | 3,222,264 | 1 | 305 |
| LG54 | 5,313,425 | 4 | 241 |
|  |  |  |  |
| Average(±SD) | 4,113,847 (±968,084) | 1.50 (±0.95) | 345.19 (±76.89) |
| Total | 222,147,733 | 81 | 18,640 |

**Supplementary Table S6** **Classification of tandem repeats in the *B. planiculmis* genome.**

| **Type** | **Number** | **Length** | **Rate(%)** |
| --- | --- | --- | --- |
| Microsatellite (1-9 bp units) | 127,116 | 3,039,892 | 1.28 |
| Minisatellite (10-99 bp units) | 41,510 | 14,208,946 | 5.97 |
| Satellite (>=100 bp units) | 7,244 | 18,996,612 | 7.98 |
|  |  |  |  |
| Total | 175,870 | 36,245,450 | 15.23 |

**Supplementary Table S7** **Classification of transposable elements in the *B. planiculmis* genome.**

| **Type** | **Number** | **Length(bp)** | **Rate(%)** |
| --- | --- | --- | --- |
| ***class Ⅰ Retroelement*** | **44837** | **25649667** | **10.7772** |
| DIRS | 3 | 211 | 0.0001 |
| LINE | 12704 | 2315379 | 0.9728 |
| LTR |  |  |  |
| Copia | 5483 | 5483827 | 2.3041 |
| ERV | 1156 | 125116 | 0.0526 |
| Gypsy | 5452 | 13264445 | 5.5733 |
| Ngaro | 137 | 9641 | 0.0041 |
| Pao | 74 | 4951 | 0.0021 |
| Unknown | 16036 | 3997233 | 1.6795 |
| SINE | 3792 | 448864 | 0.1886 |
|  |  |  |  |
| ***class Ⅱ DNA transposon*** | **95335** | **26267584** | **11.0368** |
| CACTA | 1628 | 361898 | 0.1521 |
| Crypton | 44 | 2162 | 0.0009 |
| Dada | 177 | 9620 | 0.0040 |
| Ginger | 56 | 3185 | 0.0013 |
| Helitron | 1222 | 417368 | 0.1754 |
| IS3EU | 67 | 4242 | 0.0018 |
| Kolobok | 290 | 32501 | 0.0137 |
| Maverick | 89 | 6524 | 0.0027 |
| Merlin | 121 | 6488 | 0.0027 |
| Mutator | 787 | 615958 | 0.2588 |
| P | 86 | 5197 | 0.0022 |
| PIF-Harbinger | 628 | 41527 | 0.0174 |
| PiggyBac | 33 | 1798 | 0.0008 |
| Tc1-Mariner | 109 | 6791 | 0.0029 |
| Unknown | 87905 | 24487674 | 10.2889 |
| Zisupton | 50 | 2524 | 0.0011 |
| hAT | 2043 | 262127 | 0.1101 |
|  |  |  |  |
| ***Unknown*** | 19 | 1328 | 0.0006 |
|  |  |  |  |
| **TOTAL** | **140191** | **51918579** | **21.8145** |

**Supplementary Table S8.** Summary of the gene prediction and annotation results

| **Type** | **Statistics** |
| --- | --- |
| *Gene Prediction* |  |
| Number of predicted genes | 18,760 |
| Total coding sequence length (bp) | 24,430,944 |
| Mean gene length (bp) | 3723.45 |
| Mean CDS length (bp) | 1302.29 |
| Mean number of exons per gene | 6.13 |
| Mean number of introns per gene | 5.13 |
|  |  |
| *Gene Annotation* |  |
| NR Annotated Percent (%) | 98.30 |
| Swiss-Prot Annotated Percent (%) | 83.42 |
| KEGG Annotated Percent (%) | 74.01 |
| KOG Annotated Percent (%) | 55.16 |
| TrEMBL Annotated Percent (%) | 98.66 |
| EggNOG Annotated Percent (%) | 85.42 |
| Pfam Annotated Percent (%) | 87.9 |
| GO Annotated Percent (%) | 83.96 |
| Total account of annotated genes | 18,562 |
| Total Annotated Percent (%) | 98.94 |
| *Gene prediction BUSCO* |  |
| Complete BUSCOs (%) | 94.18 |
| Complete & single-copy  BUSCOs (%) | 92.26 |
| Complete & duplicated  BUSCOs (%) | 1.92 |
| Fragmented BUSCOs (%) | 1.30 |
| Missing BUSCOs (%) | 4.52 |
| Total groups searched | 1614 |
